# Supplementary material for: Development and Molecular Cytogenetic Identification of Two Wheat-Aegilops geniculata Roth 7Mg Chromosome Substitution Lines with Resistance to Fusarium Head Blight, Powdery Mildew and Stripe Rust
Source: Int J Mol Sci. 2022 Jun 24;23(13):7056. doi: 10.3390/ijms23137056 (PMC9266563; doi:10.3390/ijms23137056)
Supplement: Supplementary file 1 [file ijms-23-07056-s001.zip › ijms-1757069-supplementary.pdf]

Supplementary Materials for

Development and Molecular Cytogenetic identification of Two Wheat-*Aegilops geniculata* Roth 7M^g Chromosome Substitution Lines with Resistance to Fusarium Head Blight, Powdery Mildew, and Stripe Rust

Xiaoying Yang[1], Maoru Xu[1], Yongfu Wang[1], Xiaofang Cheng[1], Chenxi Huang[1], Hong Zhang[1,2,3], Chunhuan Chen[1,2,3], Yajuan Wang[1,2,3,*], Wanquan Ji[1,2,3,*]

Correspondence authors: Yajuan Wang, E-mail address: wangyj7604@163.com; Wanquan Ji, E-mail address: jiwanquan2008@126.com

This word file includes:

Figures S1 to S3

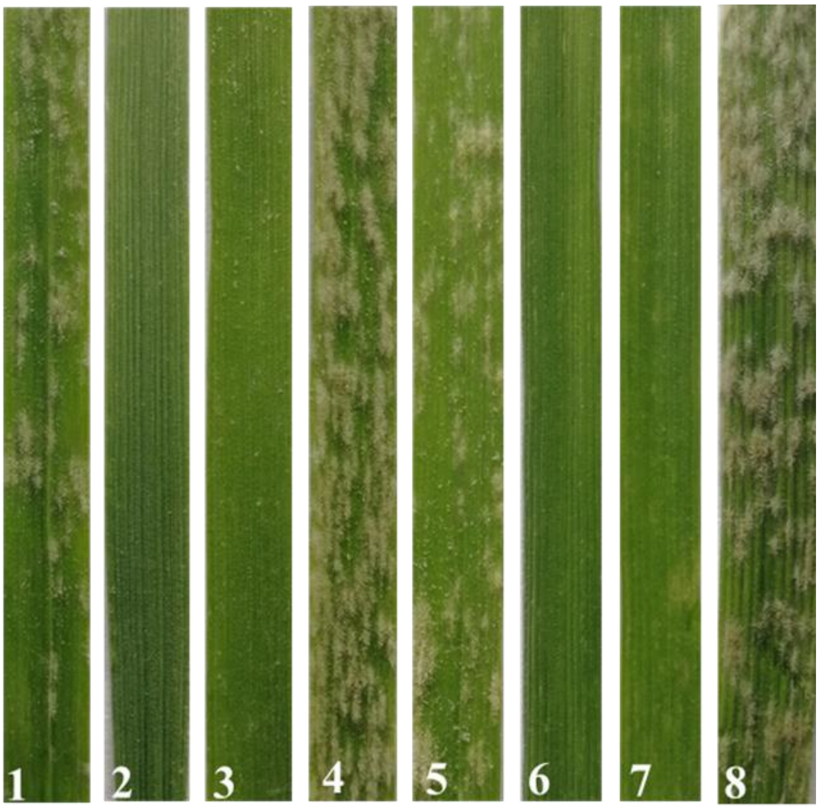

Figure S1. Leaves infected with *Blumeria graminis* at the seeding stage. 1. Triticum aestivum cv. Chinese Spring; 2. *Ae. geniculata*; 3. wheat- *Ae. geniculata* 7M^g addition line W166; 4. Abbondanza deletion line 7A; 5. Abbondanza deletion line 7B; 6. W623; 7. W637; 8. Shaanyou 225.

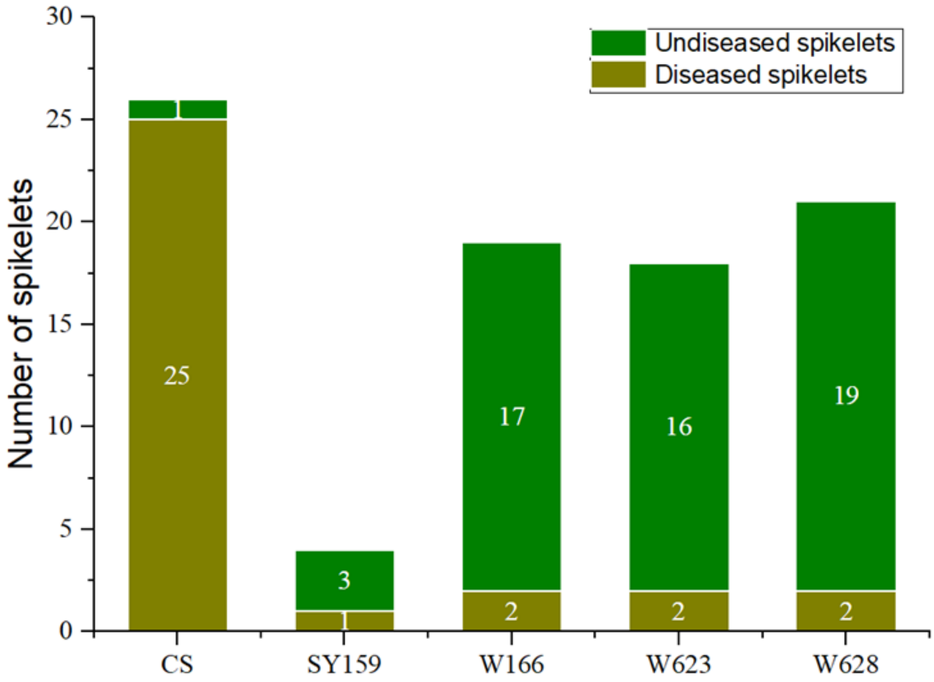

Figure S2. Number of diseased spikelets 21 days after single floret inoculation.

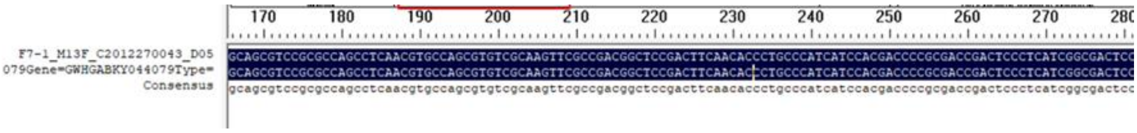

Figure S3. Sequence alignment of *GST* for development of *Fhb7* for development of the *F7-1* marker. The above sequence was obtained by sequencing the specific fragment amplified in *Th. Elongatum*, and the following sequence is the *GST* gene sequence.
